# Multiparametric monitoring of chemotherapy treatment response in locally advanced breast cancer using quantitative ultrasound and diffuse optical spectroscopy

**DOI:** 10.18632/oncotarget.7844

**Published:** 2016-03-02

**Authors:** William T. Tran, Charmaine Childs, Lee Chin, Elzbieta Slodkowska, Lakshmanan Sannachi, Hadi Tadayyon, Elyse Watkins, Sharon Lemon Wong, Belinda Curpen, Ahmed El Kaffas, Azza Al-Mahrouki, Ali Sadeghi-Naini, Gregory J. Czarnota

**Affiliations:** ^1^ Department of Radiation Oncology, Sunnybrook Hospital, Toronto, Canada; ^2^ Department of Radiation Oncology, University of Toronto, Toronto, Canada; ^3^ Department of Medical Biophysics, University of Toronto, Toronto, Canada; ^4^ Centre for Health and Social Care Research, Sheffield Hallam University, Sheffield, UK; ^5^ Division of Radiology, Sunnybrook Hospital, Toronto, Canada; ^6^ Division of Anatomic Pathology, Sunnybrook Hospital, Toronto, Canada; ^7^ Department of Nursing, Sunnybrook Hospital, Toronto, Canada

**Keywords:** diffuse optical spectroscopy, quantitative ultrasound, locally advanced breast cancer, treatment monitoring, neoadjuvant chemotherapy

## Abstract

**Purpose:**

This study evaluated pathological response to neoadjuvant chemotherapy using quantitative ultrasound (QUS) and diffuse optical spectroscopy imaging (DOSI) biomarkers in locally advanced breast cancer (LABC).

**Materials and Methods:**

The institution's ethics review board approved this study. Subjects (*n* = 22) gave written informed consent prior to participating. US and DOSI data were acquired, relative to the start of neoadjuvant chemotherapy, at weeks 0, 1, 4, 8 and preoperatively. QUS parameters including the mid-band fit (MBF), 0-MHz intercept (SI), and the spectral slope (SS) were determined from tumor ultrasound data using spectral analysis. In the same patients, DOSI was used to measure parameters relating to tumor hemoglobin and composition. Discriminant analysis and receiver-operating characteristic (ROC) analysis was used to classify clinical and pathological response during treatment and to estimate the area under the curve (AUC). Additionally, multivariate analysis was carried out for pairwise QUS/DOSI parameter combinations using a logistic regression model.

**Results:**

Individual QUS and DOSI parameters, including the (SI), oxy-hemoglobin (HbO_2_), and total hemoglobin (HbT) were significant markers for response after one week of treatment (*p* < 0.01). Multivariate (pairwise) combinations increased the sensitivity, specificity and AUC at this time; the SI + HbO_2_ showed a sensitivity/specificity of 100%, and an AUC of 1.0.

**Conclusions:**

QUS and DOSI demonstrated potential as coincident markers for treatment response and may potentially facilitate response-guided therapies. Multivariate QUS and DOSI parameters increased the sensitivity and specificity of classifying LABC patients as early as one week after treatment.

## INTRODUCTION

Breast cancer is the second leading cause of cancer-related mortalities in women and a major public health problem worldwide [1]. There are as many as 17 tumor subtypes, which are characterized by contrasting histological and molecular features [2, 3]. However, approximately 5–15% of all breast tumors will exhibit similar disease patterns that are consistent with locally advanced breast cancer (LABC); defined as stage 3–4 tumors, ≥ 5 cm in size, and may involve one or more lymph nodes [4]. Treatment typically includes pre-operative (neoadjuvant) chemotherapy (NAC), followed by definitive surgery, then radiation therapy. The major benefit to NAC is to downstage the disease to facilitate surgical resection, and to enable clinical surveillance of the tumor in response to therapy [5]. NAC has not yet been demonstrated to enhance long-term survival, but a survival benefit has been indicated in patients who attain complete pathological response in comparison to patients with progressive or residual disease after NAC [6, 7]. In general however, survival outcomes are poor; only 25% of women may achieve complete pathological response [8] and up to 46% of patients may develop recurrence within 5 years [9].

Breast cancer imaging during NAC can provide important clinical information about response to treatment, and potentially impact treatment outcomes if used to guide therapy. Current imaging methods such as x-ray mammography, and conventional computed tomography imaging can be used to study anatomical characteristics, but may be limited in showing functional and biological changes which start to occur early after treatment initiation [10]. Understanding these changes can potentially improve treatment strategies for personalized medicine. In response to these clinical challenges, imaging biomarkers have gained widespread interest in recent years as a potential tool to measure tumor response to NAC. This is due to technological improvements in imaging resolution, contrast, sensitivity and available approaches to assess pathophysiological tumor changes. Research into imaging biomarkers have aimed to match the sensitivity and specificity of gold-standard approaches from immunohistochemistry and microscopy, while providing quantitative and “real-time” results non-invasively. Emerging technologies are demonstrating that imaging biomarkers can detect important tumor characteristics such as cell death, tumor vascularity, and metabolic activity that relate to tumor response [11]. Rousseau *et al.* measured tumor metabolic activity using ^18^F-FDG-PET by quantifying the passage and clearance of radioactively labeled metabolites in solid tumors. The study demonstrated significant differences in the standard uptake value between responding/non-responding tumors during treatment [12]. Other developments in MRI-based functional imaging techniques, such as blood oxygenation-level dependent (BOLD) contrast, have indicated some promising results to measure vascular oxygenation as a marker for treatment response in tumors [13]. BOLD-MRI detects T_2_-weighted signals from deoxy-hemoglobin, and a number of studies to date have explored BOLD-MRI in breast cancer [14, 15]. For example, a pilot study by Jiang *et al.* demonstrated significant differences in tumor blood oxygen in patients with complete pathological response versus patients with partial response or stable disease to NAC [13]. Other MRI methods relating to tumor vascular perfusion use dynamic contrast-enhanced (DCE)-MRI to predict NAC treatment outcomes. Data reported on 20 LABC patients by Craciunescu *et al.* showed that predictive models with DCE-MRI parameters could classify pathological responders and non-responders with 91% sensitivity and 78% specificity [16]. Other important contributions in MRI have included diffusion-weighted imaging (DWI) to provide information on tissue microstructure related to cell death and this technique has been exploited for NAC response in breast tumors. Previous studies using DWI-MRI have demonstrated an increased accuracy in classifying patient response to NAC for breast cancer [17]. However, the routine implementation of such specialized methods from MRI and PET is limited by their invasiveness, need for contrast agents, cost, or patient exposure to ionizing radiation [18]. Additionally, other limiting factors include patient motion during imaging and the dependency on other factors such as cardiac output for contrast enhancement, which may affect data stability [13, 19]. There is recent evidence to suggest that imaging markers from quantitative ultrasound (QUS) and diffuse optical spectroscopy imaging (DOSI) can reflect early biological response to chemotherapy. Previous studies have monitored treatment using either QUS or DOSI individually, using both laboratory or commercially based imaging devices [20–23]. Both QUS and DOS imaging modalities have the advantage of being non-invasive, relatively cost effective, quick, and provide functional information about metabolism, physiology, and biological activity.

### Quantitative ultrasound

**Figure 1:**
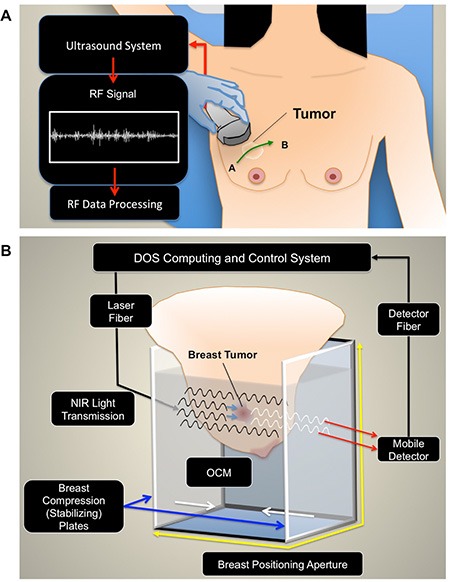
Experimental setup (**A**) QUS imaging. For QUS, patients were positioned supine for ultrasound imaging. A panoramic scan was acquired over the affected breast, which included the entire volume of the tumor, and also normal breast tissue (points A and B). (**B**) Diffuse Optical Spectroscopy Imaging. Immediately after sonography, patients were transferred onto a diffuse optical tomography device. The breast was positioned into an imaging aperture and compressed by stabilizing plates to limit motion, and maintain similar breast thickness throughout the scan series. Optical compensation fluid was added to the imaging aperture to improve light transmission between the varying surfaces.

Quantitative ultrasound uses either low or high (> 20 MHz) frequency ultrasound for tissue characterization, based on the desired acoustic resolution, and required depth for imaging. QUS uses the spectral information of radiofrequency (RF) signals that are typically discarded in conventional grey-scale sonography [Figure 1]. The spectral information of the RF signal is retained and processed by applying a Fourier transform to the signal to compute a frequency-dependent power spectrum [24]. QUS parameters, such as the mid-band fit (MBF), 0-MHz intercept (SI) and spectral slope (SS) are determined by applying a linear regression function within a discrete frequency bandwidth of the computed power spectrum [24–27]. In early studies by Lizzi *et al.*, QUS parameters were studied for therapy response monitoring in hyperthermia-treated ocular tumors [27]. The results of their study showed an increase in the SI in responsive lesions, in comparison to the surrounding normal tissue (*p = 0.003*). This increase in the backscatter intensity was explained as corresponding to changes in tissue microstructure caused by focal areas of increased cell death [27]. It was hypothesized that changes in the scattering surfaces at subcellular levels from cell death, such as fragmented nuclear structures, may modulate acoustic scattering in tissue. Later reports by Czarnota and colleagues applied Lizzi *et al.*'s theoretical framework to study the effects of apoptotic cell death and QUS in acute myeloid leukemia (AML) cells treated with chemotherapy *in vitro* [25]. That work used QUS methods as markers for apoptotic cell death. Chemotherapy-treated AML cells demonstrated a 2.92-fold to 5.83-fold increase in backscatter intensity compared to non-treated cells, and histological data revealed morphological changes resulting from cellular pyknosis, karyorhexis and apoptotic cell death [25]. In another study, Kolios *et al.* demonstrated an increase in the MBF (+13 dB) after treating AML cells to chemotherapy *in vitro*, and linked these findings to morphological changes from chromatin condensation [26]. These studies demonstrated the link between changes in tissue features, nuclear morphology and the resulting acoustic scattering in tissue [28]. Theoretical frameworks in these early QUS studies for cancer imaging have driven efforts to study chemotherapy response in breast cancer *in vivo* [20, 29]. To date, QUS has been demonstrated for functional imaging to monitor treatment response in photodynamic therapy, chemotherapy, and radiation therapy; both in animal and human studies [20, 25, 29–32].

### Diffuse optical spectroscopy

Other specialized functional imaging, such as DOSI is capable of measuring aspects related to tumor vasculature but is additionally capable of measuring other biochemical features such as water and lipid content, and tissue scattering. DOSI measures light absorption and scattering in the near-infrared spectrum (600–1100 nm) to evaluate the concentrations of endogenous chromophores such as hemoglobin, oxyhemoglobin, lipids and water, but also permitting maps of tissue oxygen-saturation to be made [33]. DOSI typically uses a large spectral bandwidth and systems can be built as handheld, or larger tomographic systems that are often referred to as diffuse optical tomography (DOT) devices. Several studies to date have utilized both research-based and commercially developed products. Both systems have their respective advantages, such as broad optical bandwidth and tissue penetrance. For example, advantages for DOT include the capability of imaging deeper tumors, and major technical advancements have increased the performance of DOT systems to separate the specific contributions of light absorption and scattering in tissue for improved tissue contrast.

Three types of DOSI techniques, such as frequency domain (FD), time domain (TD) or continuous wave (CW) have been used to measure photon migration in tissue. Continuous wave systems emit light with constant amplitude and measure the attenuation [34]; while other systems use frequency domain methods to emit light that are sinusoidally modulated at high frequencies. FD detection systems measure the attenuation and phase shift of the light to report the absorption and scattering. The major advantage to FD systems is a relatively higher signal-to-noise ratio, and can be portable, which makes it potentially desirable as a “bedside” tool. In a TD system, used in the present study, short pulses of light are emitted and the time-of-flight are measured. The major advantage is the tissue-depth penetrance and improved resolution, compared to other DOSI systems. However TD systems are often large due to the requirement for several subcomponents used in signal detection and processing.

Continuous wave, frequency domain and time domain systems utilize the absorption co-efficient to calculate the biochemical composition of tissue. Using the Beer-Lambert law, with the known molar extinction co-efficient, one can calculate the concentrations of hemoglobin, oxyhemoglobin, water, and lipids. It is important to note that breast tissue demonstrates significantly higher scattering than absorption, and this is due to the tissue's composition, and cellular structure. Other DOSI parameters such as the scatter power and scatter amplitude, calculated by using the power-law function, are representative of the tissue's substructure, which is related to cellularity, cell arrangement and light-scatterer spatial distributions [35]. As a result, DOSI can demonstrate a good sensitivity to the biochemical characteristics of tissue.

In the present study, combined QUS and DOSI parameters as predictive models, are examined to classify chemotherapy-treated LABC patients into response groups. Earlier reports from our group have indicated that QUS and DOS imaging as independent modalities, may be useful for monitoring treatment response [20, 21, 36]. A study by Soliman *et al.* studied 10 patients with DOSI, and reported a significant decrease in DOSI parameters such as oxyhemoglobin, deoxy-hemoglobin and scatter power after four weeks of treatment for responders [36]. Similarly, Sadeghi *et al.* recently demonstrated that cell death markers from QUS, such as the MBF and SI were significantly increased for responders after 4 weeks of chemotherapy [20]. Both studies demonstrated a concordance in the times that QUS and DOSI biomarkers were significant. Unlike the present study however, QUS and DOS imaging studies were independent of each other, and these reports examined separate patient cohorts. However, the results of those studies suggested (expectedly) that responding breast tumors exhibited concurrent biological markers for tumor cell death, decreased metabolism and potential vascular reorganization. Therefore, the motivation of the present study was to build on those previous reports to specifically: i) use QUS and DOSI within a single patient cohort to measure breast tumor biology during NAC and; ii) examine the possibility of QUS/DOSI multiparametric combinations to improve the classification of breast tumor response at early stages of neoadjuvant chemotherapy.

Tumor responses can be characterized by several biological events, but have three common criteria: 1) tumor cell death; 2) hematological/vascular modulation and; 3) tumor morphological changes. These criteria are closely interrelated and yet, one single factor may not completely predict patient response with high accuracy, since tumor response and resistance is a multifactorial process [37]. In the study here, it is hypothesized that building predictive models at early stages of NAC with combined QUS and DOSI parameters, may reflect these complementary, and multifactorial changes; namely by characterizing tumor cell death, vascularity, and tumor morphology concurrently. QUS/DOSI imaging was used on 22 cases of locally advanced breast cancers: 14 of which were clinical responders (R) and 8, which were non-responders (NR) and demonstrated a close correspondence between changes detected by the two different imaging modalities.

## RESULTS

**Figure 2:**
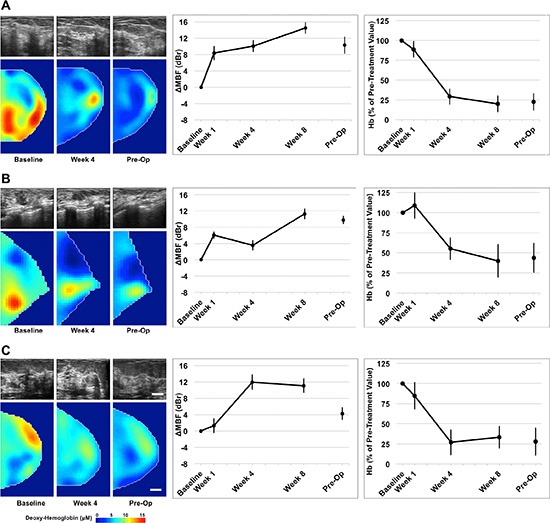
Representative QUS and DOS data of three clinical/pathological responders QUS and DOS imaging was acquired at weeks 0, 1, 4, 8, and preoperatively, relative to the start of chemotherapy. Representative B-mode images and DOSI parametric maps are presented for baseline, mid-treatment (week 4) and pre-operative scans. Responsive patients demonstrated an overall increase in QUS ultrasound backscatter intensity up to week 8, measured by the mid-band fit. These patients demonstrated a co-incident overall reduction in many DOSI parameters, such as deoxyhemoglobin (presented) during treatment. *Error bars = Standard deviation, Scale bars; US = 2 cm, DOS, 2 cm. Deoxy-hemoglobin [Hb] color bar = 0*–*15 μM. Representative figures presented correspond with Patient No. 10 (2A); No. 8 (2B); No. 6 (2C) [Table 1].*

**Figure 3:**
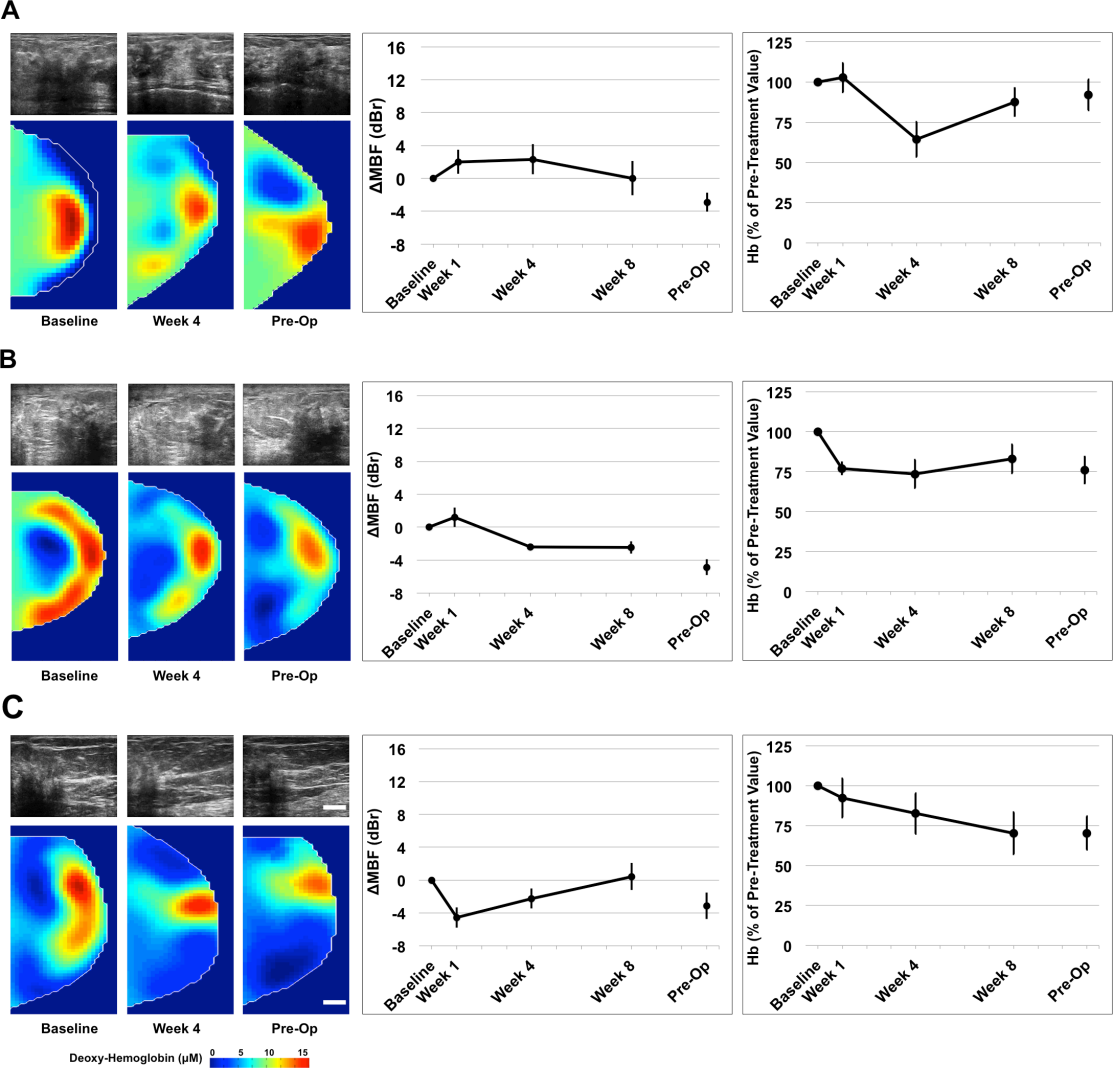
Representative QUS/DOS images and data for three non-responders Non-responsive patients demonstrated an insignificant change in the mid-band fit (dBr) and lesser changes in DOS parameters (deoxyhemoglobin presented) during treatment. *Error bars = Standard deviation, Scale bars; US = 2 cm, DOS = 2 cm. Deoxy-hemoglobin [Hb] color bar = 0*–*15 μM Representative figures presented correspond with Patient No. 22 (3A); No. 16 (3B); No. 20 (3C) [Table 1].*

Data indicated significant differences between clinical/pathological responders and non- responders with both imaging modalities. Clinical and patient characteristics are summarized in Table 1. Representative data for three responders and three non-responders are presented in Figures 2 and 3. Representative clinical characteristics and imaging findings for a typical responder and non-responder are described below.

**Table 1:** Patient and clinical characteristics

	Clinical Features				Pathologic Features				Pre-Treatment		Post-Treatment
No.	R/NR	Pre/Post Menopausal Status^1^	Laterality	Chemo Strategy	Histological Type	ER	PR	HER2	Thickness (DOT) (mm)	Tumor Size (MRI) (cm)	Tumor Size (Pathology) (cm)
1	R	Post	Right	ACT + H	IDC	+	+	+	60.0	2 × 2 × 3	0.2
2	R	Pre	Left	ACT + H	IDC	−	−	+	55.2	2 × 3 × 1	0.2
3	R	Pre	Right	ACT	IDC	+	+	−	55.8	2 × 2 × 2	0.5
4	R	Post	Right	FEC D + H	IDC	+	−	+	75.7	5 × 5 × 2	0.0
5	R	Post	Left	FEC D	IDC	+	+	−	80.4	7 × 7 × 3	1.0
6	R	Post	Right	FEC D	ILC	+	−	−	84.4	7 × 6 × 5	1.0
7	R	Pre	Right	FEC D + H	IDC	+	+	+	70.1	8 × 6 × 3	0.4
8	R	Pre	Right	ACT + H	IDC	+	+	+	80.5	5 × 4 × 5	1.6
9	R	Post	Right	ACT + H	IDC	+	+	+	60.7	2 × 3 × 3	0.2
10	R	Post	Right	ACT	IDC	+	+	−	75.3	9 × 9 × 6	2.0
11	R	Pre	Left	FEC D	IDC	+	+	−	75.0	9 × 7 × 4	0.2
12	R	Post	Left	ACT + H	IDC	+	+	+	85.5	6 × 4 × 3	0.5
13	R	Post	Right	ACT	IDC	+	+	−	85.0	9 × 6 × 7	0.5
14	R	Pre	Right	ACT	IDC	−	−	−	60.5	3 × 2 × 2	0.0
15	NR	Pre	Left	ACT	IDC	−	−	−	66.7	5 × 5 × 4	3.4
16	NR	Pre	Right	ACT	IDC	+	+	−	85.0	9 × 7 × 6	4.5
17	NR	Pre	Right	ACT	IDC	+	+	−	84.3	10 × 12 × 8	8.0
18	NR	Post	Right	ACT + H	IDC	+	+	+	75.5	8 × 7 × 3	8.4
19	NR	Post	Right	FEC D	IDC	−	−	−	68.9	5 × 2 × 1	2.8
20	NR	Post	Left	ACT	IDC	−	−	−	78.9	6 × 4 × 5	12.6
21	NR	Pre	Left	ACT	IDC	−	−	−	84.9	10 × 10	11.4
22	NR	Post	Right	ACT + H	IDC	−	−	+	65.0	5 × 4 × 2	5.0

22 Patients were studied.

*Clinical Features*: R = Responder, NR = Non-Responder, A = Adriamycin, C = Cyclophosphamide, T = Taxol, H = Herceptin, F = Fluorouracil, E = Epirubicin, D = Docetaxel.

*Pathologic Features*: IDC = Invasive Ductal Carcinoma, ILC = Invasive Lobular Carcinoma, ER = Estrogen Receptor, PR = Progesterone Receptor, HER2 = Human Epidermal Growth Factor Receptor 2.

Patients listed here were not recruited consecutively.

Pre-treatment and post-treatment measurements reported from the patient's medical records.

1 Mean age of all participants: 51 years old.

### Representative clinical/pathological responder (R)

This post-menopausal woman presented with a locally advanced breast tumor in the upper inner quadrant of the right breast that measured 9 × 9 × 6 cm by MRI. Core biopsy revealed a high-grade invasive ductal carcinoma that was estrogen receptor (ER) and progesterone receptor (PR) positive, and negative for HER2-Neu (HER2) overexpression. Neoadjuvant chemotherapy consisted of AC-T. Histological examination at the time of mastectomy revealed pathological response to treatment. Figure 2A presents representative QUS and DOSI data for this patient. After 4 weeks of treatment, this patient demonstrated an increase in the mid-band fit (Δ_MBF_) of +10.0 ± 1.4 dBr [± SD]. At the same time interval, the DOSI-measured hemoglobin concentration (Hb) decreased to 29.1% ± 9.5%, relative to the baseline. Figure 2B, 2C are presented for other responding patients.

### Representative clinical/pathological non-responder (NR)

A post-menopausal woman presented with a tumor in the right breast, which measured 5 × 4 × 2 cm by MRI. Core biopsy confirmed the presence of invasive ductal carcinoma that was confirmed ER, PR negative, and positive for HER2. Chemotherapy treatment consisted of AC-T + H. Pathological examination after mastectomy demonstrated only minimal response to neoadjuvant treatment. Representative patient data is shown in Figure 3A. This patient, in contrast to the one above, demonstrated a smaller change in the MBF at week four (ΔMBF = +2.3 ± 1.8 dBr), which was coincident with a reduction in hemoglobin to 64.4% ± 10.9% [± SD], relative to baseline values. Figure 3B, 3C are presented for other non-responding patients.

### Study data summary

#### Univariate analysis of QUS and DOSI parameters

**Figure 4:**
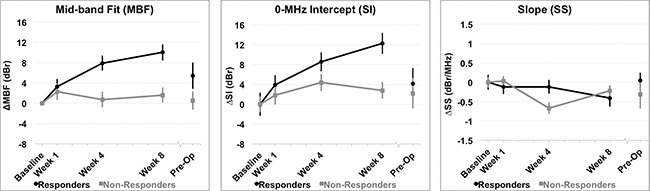
QUS parameters measured Relative changes resulting from treatment effects are presented in QUS parameters for all patients grouped by clinical response. (**A**) Mid-band Fit (MBF); (**B**) 0-MHz Intercept (SI); (**C**) Spectral Slope (SS). *Error bars = Standard deviation, n = 14 responders and n = 8 non-responders. Significant differences between responders and non-responders were tested at each time interval and parametric changes over time were tested for responders and non-responders [Table 2].*

**Figure 5:**
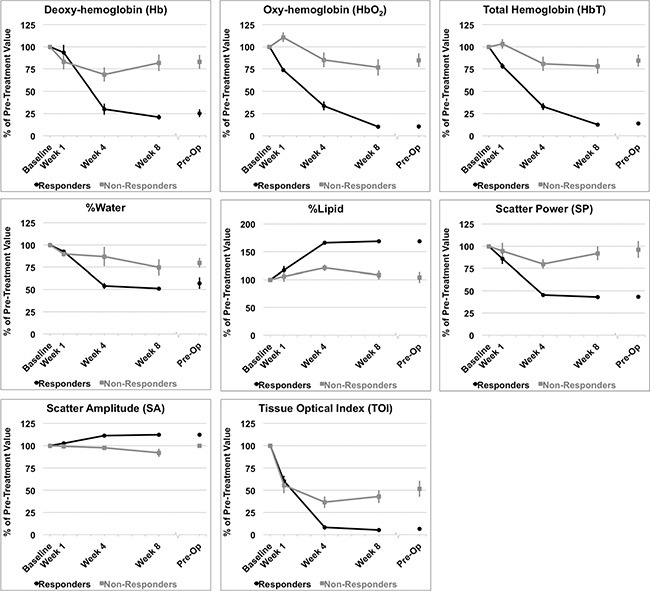
DOSI parameters measured Percent changes resulting from treatment effects according to clinical response of patients to neoadjuvant chemotherapy. Hemodynamic and tissue changes within the tumor volume are presented. *Error bars = Standard deviation, n = 14 responders and n = 8 non-responders. Significant differences between responders and non-responders were tested at each time interval and parametric changes over time were tested for responders and non-responders [Table 2].*

Significant differences between response groups were detected in estimated parameters as early as one week after the start of chemotherapy, such as the 0-MHz intercept (SI), and tumor hemoglobin parameters (HbO_2_, and HbT) (*p < 0.01*) [Table 2]. After four weeks, QUS parameters such as MBF and SI were significantly different between responders and non-responders (*p < 0.001*). This corresponded to a significant reduction in several DOSI parameters at the same experimental times such as the Hb, HbO_2_, HbT, %Water, %Lipids, SP, SA, TOI [Figures 4, 5, Table 2]. However, there were no significant differences in the SS between responders and non-responders during this time (*p > 0.05*) and this corresponded to overall insignificant changes in the spectral slope (SS) during treatment for responders (*p = 0.161*) and non-responders (*p = 0.127*). After 8 weeks, there were significant differences between groups for QUS and DOSI parameters: MBF, SI, Hb, HbO2, HbT, %Lipids, SP, SA and TOI (*p < 0.01*). At this time, QUS parameters such as the MBF increased significantly for responders (ΔMBF = 10.0 ± 1.5 [± SD] dBr), corresponding to a significant reduction in DOSI parameters such as the HbT (12.7 ± 2.2% relative to baseline). Pre-operative QUS parameters such as the SI and SS were not significantly different between groups, however there was a significant difference for all DOSI markers at this time interval (pre-op) (*p < 0.01*). Both responders and non-responders demonstrated a significant change in the tumor hemoglobin parameters (Hb, HbO_2_, HbT) during the course of treatment (*p < 0.05*). Summary data is presented in Table 2 and Figures 4 and 5.

**Table 2:** Summary of measured *p* values

	Comparison between response groups^1^				Comparison over treatment time^2^	
	Week 1	Week 4	Week 8	Pre-Op	R	NR
	*p*				*p*	
**MBF**	0.413	0.000	0.000	0.020	0.000	0.474
**SI**	0.009	0.001	0.000	0.306	0.000	0.113
**SS**	0.222	0.275	0.116	0.375	0.161	0.127
**Hb**	0.375	0.002	0.000	0.000	0.000	0.005
**HbO_2_**	0.000	0.001	0.000	0.000	0.000	0.015
**HbT**	0.004	0.000	0.000	0.000	0.000	0.003
**%Water**	0.495	0.008	0.062	0.001	0.000	0.241
**%Lipids**	0.838	0.000	0.000	0.000	0.000	0.595
**SP**	0.838	0.000	0.000	0.000	0.000	0.595
**SA**	0.410	0.002	0.002	0.001	0.000	0.170
**TOI**	0.339	0.000	0.000	0.000	0.000	0.058

A repeated-measures ANOVA was used to test for significant changes over time for QUS and DOSI parameters. QUS and DOS parameters were also tested for significant differences between responders and non-responders using an independent *t*-test within the 95% confidence level following a test for normality. Otherwise, a Mann-Whitney test was performed.

1 Independent *t*-test, tested for normality violations.

2 Repeated measures ANOVA.

*p* < 0.05, Considered statistically significant.

*p* < 0.01, Considered very statistically significant.

**Figure 6:**
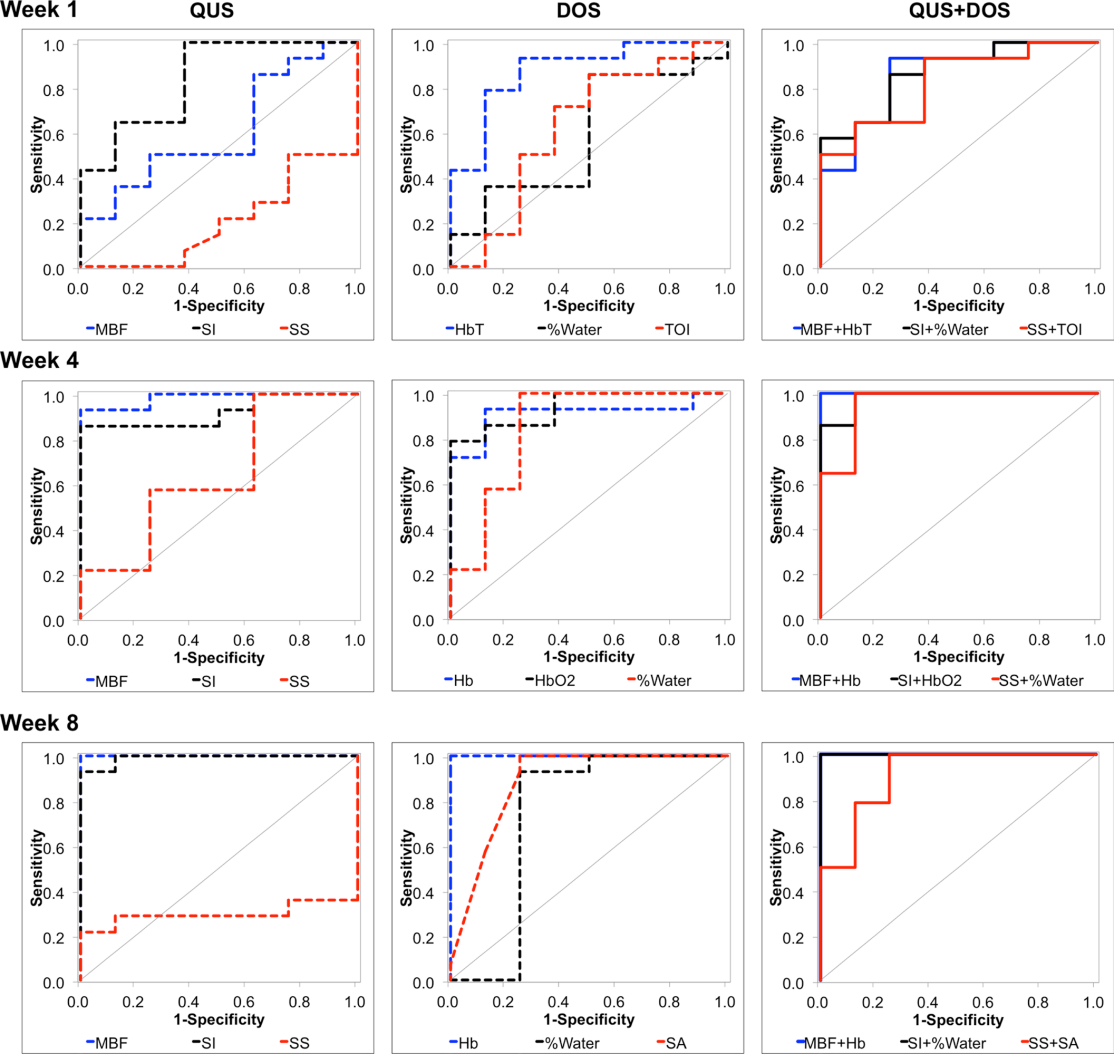
Receiver operating characteristic and corresponding area under curve ROC analysis was carried out for QUS parameters, DOS parameters, and combined pairwise combinations (QUS + DOS). All individual and pairwise combinations are summarized in Tables 3 and 4, and Supplementary Table 1. ROC analyses demonstrate that the combination of QUS and DOS parameters may increase the AUC as early as one week after the start of treatment.

Discriminant analysis and ROC analysis of individual QUS and DOSI parameters was carried out to differentiate ultimate clinical and pathological response during treatment [Table 3]. Representative AUCs for individual QUS and DOSI parameters during treatment (Weeks 1, 4, 8) are presented in Figure 6. After one week of treatment, the QUS and DOSI parameters: SI, HbO_2_, and HbT indicated good response classification (AUC range = 0.839–0.982), and this corresponded with 64.3–85.7% sensitivity, and 75.0–87.5% specificity. Other QUS and DOSI parameters were poorer predicators at this time interval, such as the SS, %Water, % Lipids, and SA [Table 3, Figure 6]. However, after four weeks of treatment, the QUS MBF and SI markers showed an increase in the AUC (range 0.920–0.982) and this corresponded with high sensitivity and specificity (range; 85.7–100.0%). DOSI parameters related to tumor hemoglobin demonstrated high sensitivity and specificity (%S *n* = 85.7%, %S *p* = 87.5%), and an AUC of 0.911–0.964. Other DOSI parameters such as the TOI demonstrated a sensitivity and specificity of 85.7% and 87.5% respectively, and an AUC of 0.973. After 8 weeks, most QUS and DOSI parameters showed further increased sensitivity, specificity, and AUC. The MBF and SI classified patients into response groups with high sensitivity and specificity (%S *n* = 92.9–100.0% %S *p* = 87.5–100.0%; AUC range = 0.991–1.0). At the same experimental time, DOSI parameters (Hb, HbO_2_, HbT, SP, %Lipids, SA, TOI) were also good predictors (%S *n* = 92.9–100%, %S*p* = 75.0%–100%, AUC = 0.884–1.0). Statistically weaker classifiers included the SI and SS at the conclusion of chemotherapy (AUC range = 0.616–0.634), but all DOSI parameters were good classifiers (%S *n* = 85.7–100.0%; %S*p* = 87.5–100.0%; AUC = 0.870–1.0).

**Table 3:** Sensitivity (%Sn), specificity (%Sp), and area under curve (AUC) for univariate QUS and DOSI parameters

	Week 1			Week 4			Week 8			Pre-Op		
Parameters	%Sn	%Sp	AUC (Logistic)	%Sn	%Sp	AUC (Logistic)	%Sn	%Sp	AUC (Logistic)	%Sn	%Sp	AUC (Logistic)
**MBF**	50.0	50.0	0.607	92.9	100	0.982	100	100	1.000	71.4	75.0	0.804
**SI**	64.3	87.5	0.839	85.7	87.5	0.920	92.9	87.5	0.991	64.3	62.5	0.634
**SS**	28.6	25.0	0.201	57.1	62.5	0.643	28.6	25.0	0.295	64.3	62.5	0.616
**Hb**	64.3	62.5	0.616	85.7	87.5	0.911	100	100	1.000	92.9	100	0.982
**HbO_2_**	85.7	87.5	0.982	85.7	87.5	0.938	100	100	1.000	100	100	1.000
**HbT**	78.6	75.0	0.875	85.7	87.5	0.964	100	100	1.000	100	100	1.000
**%Water**	50.0	50.0	0.589	85.7	75.0	0.848	71.4	75.0	0.732	85.7	87.5	0.902
**%Lipids**	50.0	50.0	0.527	92.9	87.5	0.982	100	100	1.000	100	100	1.000
**SP**	50.0	50.0	0.527	92.9	87.5	0.982	100	100	1.000	100	100	1.000
**SA**	57.1	50.0	0.393	85.7	87.5	0.897	92.9	75.0	0.884	85.7	87.5	0.870
**TOI**	64.3	62.5	0.625	85.7	87.5	0.973	100	100	1.000	100	100	1.000

QUS and DOSI parameters were analyzed for weeks 1, 4 and 8 to correspond to response monitoring during treatment. Markers for response classification were detected as early as one week relative to the start of chemotherapy.

### Multivariate analysis of pairwise Qus/Dosi parameter combinations

Table 4 and Figure 6 represent results of the discriminant and ROC analyses of QUS/DOSI pairwise combinations during chemotherapy (Weeks 1, 4, 8). All combinations that demonstrated an AUC > 0.8 are given in Supplementary Table 1. Parametric combinations increased the sensitivity and specificity for response classification as early as one week after treatment. At week 1, combining the SI and tumor hemoglobin parameters (HbO_2_ and HbT) demonstrated a sensitivity and specificity of 85.7–100.0%. This corresponded with an AUC of 0.929–1.0. Also, combining the SS + HbO_2_ enhanced the AUC (AUC = 1.0) compared to those single parameters individually. At week 1, the combination of the MBF with tumor hemoglobin demonstrated good AUC values (range 0.857–0.973), but this did not demonstrate a classification benefit compared to using those individual parameters alone.

**Table 4:** Sensitivity (%Sn), specificity (%Sp) and AUC for representative multivariate (pairwise) QUS and DOSI parameters

Combined Parameters	%Sn	%Sp	AUC (Logistic)	*p*
Week 1				
**MBF + HbO_2_**	85.7	87.5	0.973	0.000
**MBF + HbT**	71.4	75.0	0.857	0.006
**SI + HbO2**	100	100	1.000	0.000
**SI + HbT**	85.7	87.5	0.929	0.001
**SI + %Water**	71.4	75.0	0.866	0.005
**SS + HbO_2_**	100	100	1.000	0.000
**SS + HbT**	85.7	87.5	0.955	0.000
**SS + TOI**	64.3	62.5	0.821	0.014
**Week 4**				
**MBF + Hb**	100	100	1.000	0.000
**MBF + HbO_2_**	100	100	1.000	0.000
**MBF + %Water**	100	100	1.000	0.000
**SI + SA**	100	100	1.000	0.000
**SI + HbO_2_**	85.7	87.5	0.982	0.000
**SI + %Water**	85.7	87.5	0.964	0.000
**SS + SA**	100	100	1.000	0.000
**SS + TOI**	92.9	87.5	0.982	0.000
**SS + %Water**	85.7	87.5	0.955	0.000
**Week 8**				
**MBF + Hb**	100	100	1.000	0.000
**MBF + %Lipids**	100	100	1.000	0.000
**MBF + TOI**	100	100	1.000	0.000
**SI + HbT**	100	100	1.000	0.000
**SI + %Water**	100	100	1.000	0.000
**SI+SA**	100	100	1.000	0.000
**SS + HbT**	100	100	1.000	0.000
**SS + SA**	78.6	75.0	0.911	0.002
**SS + %Water**	71.4	75.0	0.893	0.003

Pairwise combinations were reported with AUC > 0.8. Analyses were performed at week 1, 4 and 8 for combined parameters (i.e. during treatment). Combining QUS and DOSI parameters demonstrated an increase in sensitivity and specificity as early as one week after treatment. Combination pairs were analyzed using a logistic regression model, and the AUC was estimated using a receiver-operating characteristic. A complete list of all pairwise combinations (AUC > 0.8) are presented in Supplementary Table 1.

At week 4, response classification was enhanced when the MBF, SI, and SS were combined with the following DOSI parameters: Hb, HbO_2_, %Water, SA, TOI. The combination of the MBF and Hb, or HbO_2_, or %Water resulted in an AUC of 1.0, and a sensitivity and specificity of 100% [Figure 6]. The SI showed an increase in sensitivity and specificity when combined with either HbO_2_, %Water, or SA (%Sn, %S*p* = 85.7–100%). Lastly, the SS showed an improvement with combinations with the SA, or TOI or %Water (%Sn/%S *p* = 85.7–100%, AUC = 0.955–1.0). Other pairwise combinations demonstrated an increase in the sensitivity and specificity, and AUC after eight weeks of chemotherapy, compared to univariate predictors. Combinations involving the SI, showed an increase in the AUC when combined with %Water or the SA. The SI+%Water or SA resulted in an AUC of 1.0.

### Histology

**Figure 7:**
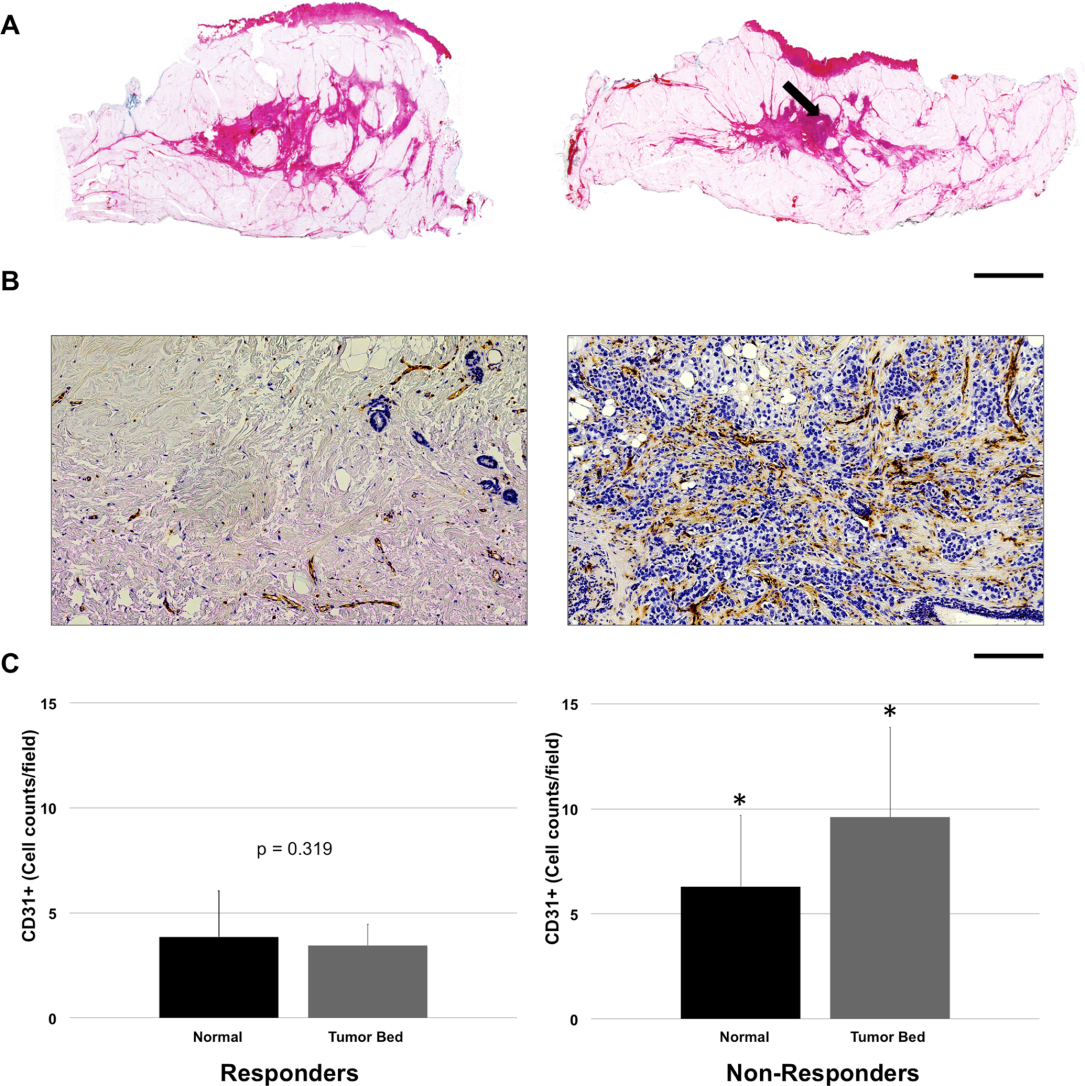
Representative comparison of responder and non-responder based on pathological examination (**A**) Reduction in the bulk tumor size in responder (left) and minimal reduction in non-responder (right, arrow); H & E stain, scale bar = 2.0 cm. (**B**) Reduction in vascular density within the tumor in responder (left) as compared to non-responder (right); CD31 immunostain, *Scale bar = 200* μ*m*. (**C**) *Vessel quantification*: For non-responders, there was significantly higher vascular staining in the tumor bed compared to normal breast tissue (*p < 0.05*). In contrast for responders, vessel counts per field showed insignificant differences (*p = 0.319*) between the tumor bed and normal tissue. *Error bars = Standard deviation*. *p < 0.05, within group comparison.

Gross examination of the tumor bed using hematoxylin and eosin staining demonstrated a reduction in the bulk tumor in responsive tumors in comparison to non-responsive patients [Figure 7A]. Post-mastectomy histology demonstrated changes in vascular density between responders and non-responders by CD31 staining [Figure 7B, 7C]. For CD31 staining, there was a significant difference in endothelial cell count within the tumor bed (*p* < 0.05) between groups, with responders showing a lower mean cell count (cells/field = 3.5 ± 1.8) in contrast to non-responders (cells/field = 9.6 ± 4.3) [Figure 7B, 7C]. After treatment, CD31 vessel counts in responding patients demonstrated insignificant differences (*p = 0.319*) between the tumor bed and normal tissue. In contrast, non-responding patients maintained a higher CD31 count in tumors when compared to non-tumor tissue (*p* < 0.05) [Figure 7C].

## DISCUSSION

In the present study, we examined combined analysis of QUS/DOSI parameters to predict NAC response in locally advanced breast cancer. DOSI parameters have been useful in demonstrating biochemical, hematological and morphological changes with treatment [23, 38, 39], while QUS parameters have demonstrated cell death in samples from chemotherapy [20, 26]. These parameters have also been tied to pathologic characteristics and outcomes [20, 22, 29, 33]. Recent work by Cerussi *et al.*, used DOSI to measure tumor water content, and tumor hemoglobin concentration at multiple times during chemotherapy treatment [33]. The results of that study indicated a significant reduction in %Water and tumor hemoglobin at the conclusion of chemotherapy when compared to the baseline measurements, and this corresponded to patients who demonstrated complete pathological response [33]. The results in the study here are consistent with their findings and in addition, DOSI data is supplemented with QUS biomarkers (MBF, SI) that indicated an increase in cell death within the tumor region. Specifically, after four weeks, the MBF and SI increased +7.9 ± 1.4 dBr and +8.5 ± 1.8 dBr, respectively in responders, which contrasted with non-responders who demonstrated a smaller intensity change of +0.7 ± 1.4 dBr (MBF) and +4.4 ± 1.6 dBr (SI). At the same time, the total hemoglobin (HbT) in responders reduced to 32.8 ± 4.0% in comparison to non-responders that showed a decrease to only 80.7% ± 8.1% from the baseline value. A possible explanation in responders could be due to decreased vessel viability within the tumor [11, 40]. Conversely, non-responding patients may have tumors with more aggressive tumor cells that prompt blood vessel growth to support metabolic demands.

In principle, tumor metabolic information, reflected by markers for deoxy-hemoglobin and oxy-hemoglobin parameters is closely linked to tumor cellular activity [21, 41]. This is explained by the conversion of oxyhemoglobin to deoxyhemoglobin during tumor cell cycling, and activity. After 8 weeks of treatment, responders demonstrated a significant reduction of [HbO_2_], and [Hb] to 10.2 ± 2.3% and 21.0 ± 2.9% of the pre-treatment values, respectively. This corresponded with an increase in MBF of +10.0 ± 1.5 dBr suggesting a coincident increase in dying cells within the tumor bed. Non-responders however, demonstrated less significant decreases in the tumor hemoglobin, relative to the pre-treatment value (76.7 ± 9.2% [HbO_2_]; 81.9 ± 9.4% [Hb]) and this was also correlated to a lesser change in the MBF and SI (+1.6 ± 1.4 dBr and +2.8 ± 1.6 dBr, respectively). Together, QUS and DOSI data suggest that chemotherapy-responsive tumors decrease in metabolism as linked to blood-based parameters in comparison to non-responding tumors; potentially as a result of dying tumor cells. QUS parameters, such as the SI and SS were not significantly different between responders and non-responders at the pre-operative time-point. This was expected since QUS measurement are sensitive to cell death induced by treatment which occurs in responsive patients early on, rather than many months later after chemotherapy. Pre-operative measurements were obtained 4–6 weeks after the last chemotherapy infusion and therefore at this time, cell death is expected to diminish within the tumor bed in responsive patients, due to a large reduction in tumor cells after many months of chemotherapy.

Tumor structure was further characterized by measuring the tissue optical index (TOI) using DOSI [35, 42]. The TOI accounts for the ratio between %Water, lipid content and deoxyhemoglobin and reflects the optical properties of breast tumors in reference to its pathological state [35]. In the work here, the TOI demonstrated significant differences (*p = 0.000*) between responders and non-responders after four weeks of chemotherapy (8.1 ± 1.9%, and 36.5 ± 6.5%, respectively from the baseline values). The TOI also differed significantly *(p < 0.001)* at the conclusion of chemotherapy and this was consistent with previous reports [33, 36, 43]. The change in TOI is dependent on the water fraction and lipid content *(Equation 2)*, and thus responsive tumors that demonstrate a larger reduction in [H_2_O] will also result in a diminished TOI value [35, 44]. Cerussi *et al.* previously suggested that this reduction in water fraction in responsive tumors might represent variations in tumor cell density, and cellularity within the tumor bed [22], and this was supported by clinically reported histological data that demonstrated cellular changes in the tumor after NAC. Although the relationship between water fraction and tumor cellularity is not entirely clear; it may be related to inflammatory response mechanisms within the tumor parenchyma [45]. Further, it was noted in the work here that %Lipid also increased for responders, which can affect the TOI. The increase in lipid content within the tumor bed could represent the changes in lipid composition closer to that of normal breast tissue.

QUS/DOSI combined parameters enhanced chemotherapy response classification in comparison to single modality parameters as early as one week after the start of NAC. However, we note that not all combinations increased the sensitivity and specificity of response assessment, and this could likely be caused by the relatively small sample size in this first study. Many single parameters classified patients with higher accuracy at weeks 4 and 8. This is likely due to the cumulative effects of treatment and the concurrent biological changes in tumors at those times. However, some parameters such as the SS benefited from multivariate QUS/DOSI combinations. It was expected that combining highly sensitive and/or specific parameters would increase the accuracy and prediction of treatment outcomes. In contrast, weaker predictors (such as the SS) would benefit from pairing with stronger predictive parameters with increases in sensitivity and specificity because more parameters carry complementary information about tumor physiology or cell death. The results of this study suggest that QUS/DOSI pairwise combinations may be useful for clinical application when modeled at one week of NAC treatment, using a combination of parameters that include: SI, SS, HbO_2_, HbT, SP, SA, %Water, and TOI, since many of these parameters demonstrate poor sensitivity and specify on their own at that time. This may potentially be followed by treatment response verification and validation by using several other QUS/DOSI parameters at weeks 4 and 8.

Other strategies for combined systems include US-guided optical imaging, developed by Zhu *et al.*, from the University of Connecticut [40, 46, 47]. These systems have been studied to measure NAC response in breast tumors. US grey-scale imaging was used there to localize breast tumors, and optical tomography to map tumor hemoglobin changes during treatment [40, 47]. The technical benefits of that approach use co-registered US images to verify posterior (deeper) tumor margins, where optical image resolution is poorer [40]. For the current study, conventional US and DOS images were not co-registered since there were differences in the spatial geometries of the QUS and DOS images but averaged values over the tumor volume were used. This was due to patient positioning for each scan modality (i.e. supine vs. prone), and breast shape from DOSI breast compression. Another study from Ueda *et al.* used multivariate analyses for baseline DOSI parameters combined with tissue biomarkers from immunohistochemistry [11]. Markers for cell proliferation (Ki67), and molecular features (estrogen and progesterone receptor) were combined with optical measurements such as [HbO_2_], [Hb], or the tumor oxygen saturation (StO_2_). Multivariate analysis of the combined parameters demonstrated an increase in the sensitivity and specificity for predicting NAC response. The results of this study from Ueda *et al.* support the need for further exploration into combination analysis to improve the predictive performance of multiple imaging and clinical biomarkers [11].

This cohort represents a first in terms of patients imaged with QUS and DOSI, probing tumor response with parallel modalities. However, limitations here included a relatively small study cohort (*n* = 22), which could potentially contribute to overestimated measures. Also, due to the small number of patients included in this report, the discriminant model was not cross validated. There were also positional limitations from using both QUS and DOSI. However to address this, we applied a volumetric analysis throughout the scan series to measure the average volumetric changes over time. Also, clinical response was confirmed using pathological endpoints and for the purpose of statistical analysis; patients were classified into binary categories (responder vs. non-responder). In practice, pathological response is graduated, and classified using standard conventions such as, Miller-Payne criteria [48]. Larger cohort studies would be needed to stratify patients, similar to pilot studies by Zhu *et al.* [47]. Pathological endpoints were also used to make inferences about the biological measurements during treatment. A major limitation was that it was not possible to validate mid-treatment tumor biology histologically, with repeat biopsies of the breast. A potential future endeavor would be to include other quantitative imaging methods, such as BOLD-MRI, and DWI-MRI to complement the physiological inferences made in this current study. The study population here demonstrated variations in chemotherapy regimens, and a patient cohort with differing molecular and histological breast cancer subtypes. However, all patients received anthracycline- or taxane-based chemotherapies, and these agents have been recognized to initiate cell death in tumors from broad categories of patients [49, 50]. With these limitations, these initial results serve as a framework for future studies into evaluating breast tumor response to NAC using combined QUS and DOSI parameters.

## CONCLUSIONS

QUS/DOS imaging biomarkers were studied in breast cancer patients to report first accounts of coincident expression during neoadjuvant chemotherapy. We observed that cell death markers from QUS are associated with a decrease in tumor hemoglobin markers from DOSI, suggesting that increased cell death and vascular remodeling are typically predictive of a favorable treatment response. Using these imaging modalities together and deriving combined acoustic and optical spectral data could provide more powerful imaging signatures to help guide treatment decisions and improve outcomes for patients. With further validation studies, it would be plausible to use QUS/DOSI markers as biological surrogates to predict tumor response to neoadjuvant chemotherapy. These imaging modalities are cost effective, non-invasive and can be acquired quickly and efficiently within the patient's treatment schedule. Co-incident QUS and DOS changes are important to understand the pathophysiological traits in tumors for better treatment response evaluation.

## MATERIALS AND METHODS

### Patient recruitment and treatment

Women were enrolled into the study (*n* = 22) following institutional ethics approval, and written informed consent was obtained from each study participant. Subjects were selected based on disease and clinical criteria, which included: LABC diagnosis, older than 18 years, and receiving neoadjuvant chemotherapy as first line standard treatment (adriamycin (A), cyclophosphamide (C) and paclitaxel (T) [AC-T] or with flouricil (F), epirubicin (E), cyclophosphamide (C) and docetaxel (D) [FEC-D]. In addition patients with Her2-Neu amplified tumors received Herceptin (H) after 3–4 cycles of treatment. Clinical and patient-specific information are summarized in Table 1. Participants were imaged at the following times relative to the start of chemotherapy: weeks 0 (baseline), one, four, eight and a final scan several weeks later prior to surgery; all patients underwent mastectomy.

### Instrumentation and imaging

Breast imaging used QUS and DOSI in sequence, to acquire both types of data using methods described previously [20, 21, 51]. For ultrasound, a continuous panoramic scan was performed on the affected breast, which included both normal breast tissue and the entire tumor volume [Figure 1A]. This was repeated for each time point throughout the imaging series for data reproducibility. A Sonix RP system (Ultrasonix, Vancouver, Canada) operating with an L14-5/60 transducer was used to collect conventional brightness mode (B-mode) and RF data (center frequency of 7 MHz, 40 MHz 8-bit dynamic range RF digitization frequency). The sector size was kept constant (lateral distance = 6 cm, axial depth 4 cm), and the focal depth was placed to correspond to the tumor's position. The focal depth remained constant throughout the ultrasound imaging series. The total duration of the ultrasound scans were approximately 20 minutes.

Immediately after sonography, DOSI data was collected. The patient was transferred onto a commercially developed diffuse optical tomography system (SoftScan, Advanced Research Technologies, Montreal, Canada). The patient was positioned prone, and the breast was placed into an enclosed imaging aperture and stabilized by opposing Plexiglas plates with soft compression in the cranio-caudal direction [Figure 1B]. The distance (thickness) between plates was recorded at baseline (average thickness = 73.3 ± 10.3 mm) and maintained during the imaging series. Optical compensation medium (OCM) was added into the imaging aperture and filled to cover the entire breast surface [Figure 1B]. The OCM was used to improve light transmission between surfaces, and was formulated as an emulsion of lipids, water, and dye to mimic optical properties of breast tissue (μ_a_= 0.05 cm^−1^ and μ_s_ =11 cm^− 1^, (λ) = 780 nm [51, 52]. The optical mammography system employed time-domain methods for imaging, and used four individual semiconductor diode lasers (LDH-P, PicQuant, Berlin, Germany) that operated at 690, 730, 780, 830 nm. The pulse duration at the full width half maximum was less than 150 ps, driven at 20 MHz. For the detection system, the light was collected using a photomultiplier (H7422P-50, Hamamatsu Photonics, Shizuoka, Japan), which was opposite to the light source. Images were reconstructed into tomographic and parametric maps of the optical parameters. Each voxel size was 3 mm × 3 mm × 7 mm^3^. The total duration of the optical mammography scan was approximately 30–40 minutes.

### Image analysis

Tumor volume analyses were carried out with the average of QUS and DOSI parameters calculated over the volume of the tumor. Selection of the region of interest (ROI) and imaging analysis was completed with the assistance of a radiologist. For the estimation of QUS parameters, spectral analysis of the tumor RF signal was performed. These methods were previously described by Lizzi *et al.* [53], and were adapted for this study. Analysis was performed using a MATLAB-based software (Matlab, Mathworks, Natick MA, USA) developed by Oelze *et al.* from the University of Illinois and previously used by our group [20]. RF Data was analyzed and averaged from 10–14 equally spaced scan planes by applying a fixed-sized ROI spanning the volume of the tumor [20]. The ROI dimensions were kept constant for the duration of the scan series, and determined at baseline. In order to reduce spectral-noise artifacts, a sliding window algorithm was used with the settings of a Hamming window function for gating, where there was an 80% overlap between adjacent windows in the axial direction. A reference phantom technique was used to remove system transfer effects from the data using a tissue-mimicking agar-embedded glass-bead phantom, with measured acoustic properties [29]. The normalized power spectrum (dBr) was calculated by dividing the power spectrum for samples by the reference power spectrum of the phantom. A linear regression line of the normalized power spectrum over the −6 dB bandwidth of the transducer was analyzed to determine the mid-band fit (MBF), 0-MHz intercept (SI) and the spectral slope (SS) [54].

For DOSI, the measured absorption and scattering coefficients were employed to calculate the DOSI parameters. The hemoglobin (Hb) and oxyhemoglobin (HbO_2_) concentrations (units; μM), and water content (%Water) were calculated using the Beer-Lambert law, given the known molar extinction coefficients for each chromophore, and by using the measured absorption coefficients. Scattering properties of breast tissue at the near-infrared light range were approximated to Mie scattering in tissue [55]. The scatter power (SP) and scatter amplitude (SA) were calculated by the power-law fit of the reduced scattering coefficients for each transmitted wavelength, expressed as:

μs = a(λ)^−b^, where *a* = scatter amplitude, *b* = scatter power *(Equation 1).*

The total hemoglobin concentration (HbT, units; μM) was calculated using the combined concentrations of oxy-hemoglobin and deoxy-hemoglobin [HbT = HbO_2_ + Hb]. Since the absorption peaks for lipids were beyond the spectral range of the imaging system, lipid content (%Lipid) was estimated using the linear relationship with the scatter power, previously described by Cerussi *et al.*, and Intes *et al.* [10, 51]. Lastly, the TOI has been defined in a variety of ways [42, 56, 57] and previously described by Cerussi *et al.* to give maximum tissue contrast in breast as:

$$\frac{[\%\text{Water}]\times \text{Hb}}{\left[\%\text{Lipid}\right]}(\text{Equation }2)[42].$$

Diffuse optical images were reconstructed with a voxel resolution of 3 mm × 3 mm × 7 mm, and then analyzed in-plane using the manufacturer's software (ART Review Workstation, V. 1.01.01, Advanced Research Technologies, Montreal, Quebec Canada). For the reconstructed images, a fixed size region of interest was selected around the tumor bed, based on TOI parametric images, and with the assistance of the patient's medical imaging record, such as MRI. The ROI included the tumor volume and the signal threshold values for analysis were determined before the start of treatment (baseline). The threshold value was kept constant for each patient throughout the analysis of all imaging series.

### Definition of response to treatment

Patients were classified as responders (R) or non-responders (NR) based on combined data from pre- and post therapy imaging and particularly pathology examination of post-treatment tissue. The pre- and post therapy imaging measurements of tumor size were performed according to RECIST 1.1 guidelines using MRI [58]. Also, as part of the standard clinical cancer treatment, a breast pathologist examined mastectomy specimens macroscopically and microscopically for any residual tumor. Specimens were stained using standard hematoxylin and eosin (H & E) techniques and examined by a board-certified staff pathologist. The responders were defined in this study as having complete pathological response or a greater than 50% decrease in tumor size as compared to pre-therapy. Non-responders were classified as having stable or progressive disease and where there was < 50% decrease in tumor size [59]. Pathological data was collected from the pathology report from the patients' medical record.

In addition, post-mastectomy CD31 immunostaining (JC07 clone, Leica Biosystems, Concord, Ontario Canada) was used to quantitatively assess vascular density within the tumor bed and adjacent mammary tissue (normal tissue) in responders and non-responders (TissueScope, Huron Digital Pathology, Waterloo, Canada). The analysis ROIs were dependent on the patient's response to treatment due to the size variance of the residual tumor bed. Stained vessels were counted for each specimen and the vessel counts were averaged across all respective normal or tumor regions analyzed.

### Statistical analysis

Descriptive statistics were used on both QUS and DOS parameters (SPSS Inc., Chicago IL, USA). The means and standard deviations were calculated for each imaging dataset taken at each time-point. For QUS, the change [Δ] in QUS parameters was calculated by subtracting the measurements at each time interval from the value measured at baseline. DOSI measurements were expressed in percent changes from the baseline [% Change]. Significant changes over time were tested for each QUS and DOSI parameter to compare its difference to baseline values using a repeated-measures ANOVA.

Independent QUS and DOS parameters were tested for significant differences between responders and non-responders at each time interval. A normality violation was tested for each parameter using a Shapiro-Wilk test. For normally distributed parameter changes, an independent *t*-test was used (unpaired, two-sided, 95% confidence interval). Otherwise, an unpaired, Mann-Whitney *U*-test within the 95% confidence level was utilized (SPSS Inc., Chicago IL, USA) for parametric changes that were not normally distributed. Discriminant analysis (SPSS Inc., Chicago IL, USA), and receiver-operating characteristic (ROC) analysis (SPSS Inc., Chicago IL, USA) estimated sensitivity and specificity, and the area under the curve (AUC) values for QUS and DOSI parameters individually. Additionally for multivariate analysis, a logistic regression model was employed to estimate the sensitivity and specificity, and AUC of combined QUS and DOSI parameters within a 95% confidence interval. The pairing/combination strategy analyzed all pairwise combinations between QUS and DOSI parameters during treatment (weeks 1, 4 and 8, relative to the start of chemotherapy). Pairwise combinations were reported for AUC classification scores that were greater than 0.8 [60]. Parameters and statistical measures were considered significant, at an alpha level of 0.05 or less.

## SUPPLEMENTARY MATERIALS TABLES


