# Supplementary material for: Multiparametric monitoring of chemotherapy treatment response in locally advanced breast cancer using quantitative ultrasound and diffuse optical spectroscopy
Source: Oncotarget. 2016 Mar 2;7(15):19762–80. doi: 10.18632/oncotarget.7844 (PMC4991417; doi:10.18632/oncotarget.7844)
Supplement: Supplementary file 1 [file oncotarget-07-19762-s001.pdf]

Multiparametric monitoring of chemotherapy treatment response in locally advanced breast cancer using quantitative ultrasound and diffuse optical spectroscopy

Supplementary Materials

Supplementary Table S1A: Sensitivity (%Sn), Specificity (%Sp) and AUC for multivariate (pairwise) QUS and DOSI parameters with AUC > 0.8

		Week 1		
**Combined Parameters**	**%Sn**	**%Sp**	**AUC (Logistic)**	***p***
**MBF + HbO_2**	85.7	87.5	0.973	0.000
**MBF + HbT**	71.4	75.0	0.857	0.006
**SI + Hb**	64.3	62.5	0.839	0.009
**SI + HbO_2**	100	100	1.000	0.000
**SI + HbT**	85.7	87.5	0.929	0.001
**SI + %Water**	71.4	75.0	0.866	0.005
**SI + %Lipids**	71.4	62.5	0.857	0.006
**SI + SP**	71.4	62.5	0.857	0.006
**SI + SA**	78.6	75.0	0.830	0.012
**SI + TOI**	78.6	75.0	0.839	0.009
**SS + HbO_2**	100	100	1.000	0.000
**SS + HbT**	85.7	87.5	0.955	0.000
**SS + %Water**	78.6	75.0	0.848	0.008
**SS + %Lipids**	78.6	75.0	0.830	0.012
**SS + SP**	78.6	75.0	0.830	0.012
**SS + SA**	78.6	75.0	0.857	0.006
**SS + TOI**	64.3	62.5	0.821	0.014

Supplementary Table S1B:

		Week 4		
**Combined Parameters**	**%Sn**	**%Sp**	**AUC (Logistic)**	***p***
**MBF + Hb**	100	100	1.00	0.000
**MBF + HbO_2**	100	100	1.00	0.000
**MBF + HbT**	100	100	1.00	0.000
**MBF + %Water**	100	100	1.00	0.000
**MBF + %Lipids**	100	100	1.00	0.000
**MBF + SP**	100	100	1.00	0.000
**MBF + SA**	100	100	1.00	0.000
**MBF + TOI**	100	100	1.00	0.000

**SI + Hb**	85.7	87.5	0.955	0.000
**SI + HbO_2**	85.7	87.5	0.982	0.000
**SI + HbT**	85.7	87.5	0.982	0.000
**SI + %Water**	85.7	87.5	0.964	0.000
**SI + %Lipids**	100	100	1.000	0.000
**SI + SP**	100	100	1.000	0.000
**SI + SA**	100	100	1.000	0.000
**SI + TOI**	85.7	87.5	0.982	0.000
**SS + Hb**	92.9	100	0.955	0.000
**SS + HbO_2**	85.7	87.5	0.938	0.001
**SS + HbT**	85.7	87.5	0.955	0.000
**SS + %Water**	85.7	87.5	0.955	0.000
**SS + %Lipids**	100	87.5	0.991	0.000
**SS + SP**	100	87.5	0.991	0.000
**SS + SA**	100	100	1.000	0.000
**SS + TOI**	92.9	87.5	0.982	0.000

Supplementary Table S1C:

		**Week 8**		
**Combined Parameters**	**%Sn**	**%Sp**	**AUC (Logistic)**	***p***
**MBF + Hb**	100	100	1.00	0.000
**MBF + HbO_2**	100	100	1.00	0.000
**MBF + HbT**	100	100	1.00	0.000
**MBF + %Water**	100	100	1.00	0.000
**MBF + %Lipids**	100	100	1.00	0.000
**MBF + SP**	100	100	1.00	0.000
**MBF + SA**	100	100	1.00	0.000
**MBF + TOI**	100	100	1.00	0.000
**SI + Hb**	100	100	1.00	0.000
**SI + HbO_2**	100	100	1.00	0.000
**SI + HbT**	100	100	1.00	0.000
**SI + %Water**	100	100	1.00	0.000
**SI + %Lipids**	100	100	1.00	0.000
**SI + SP**	100	100	1.00	0.000
**SI + SA**	100	100	1.00	0.000
**SI + TOI**	100	100	1.00	0.000
**SS + Hb**	100	100	1.00	0.000
**SS + HbO_2**	100	100	1.00	0.000
**SS + HbT**	100	100	1.00	0.000
**SS + %Water**	71.4	75.0	0.893	0.003
**SS + %Lipids**	100	100	1.00	0.000

**SS + SP**	100	100	1.00	0.000
**SS + SA**	78.6	75.0	0.911	0.002
**SS + TOI**	100	100	1.00	0.000

Pairwise combinations were reported with AUC > 0.8. Analyses were performed at week 1, 4 and 8.
